# Persian Medicine Network (PMN) Establishment in the Universal Scientific Education and Research Network (USERN)

**DOI:** 10.31661/gmj.v12i.3066

**Published:** 2023-08-23

**Authors:** Babak Daneshfard, Ebrahim Khadem

**Affiliations:** ^1^ Chronic Respiratory Diseases Research Center, National Research Institute of Tuberculosis and Lung Diseases (NRITLD), Shahid Beheshti University of Medical Sciences, Tehran, Iran; ^2^ Persian Medicine Network (PMN), Universal Scientific Education and Research Network (USERN), Tehran, Iran; ^3^ Department of Persian Medicine, School of Persian Medicine, Tehran University of Medical Sciences, Tehran, Iran

**Keywords:** Biomedical Research, Integrative Medicine, Persian Medicine, USERN

## Dear Editor,

**Figure-1:**
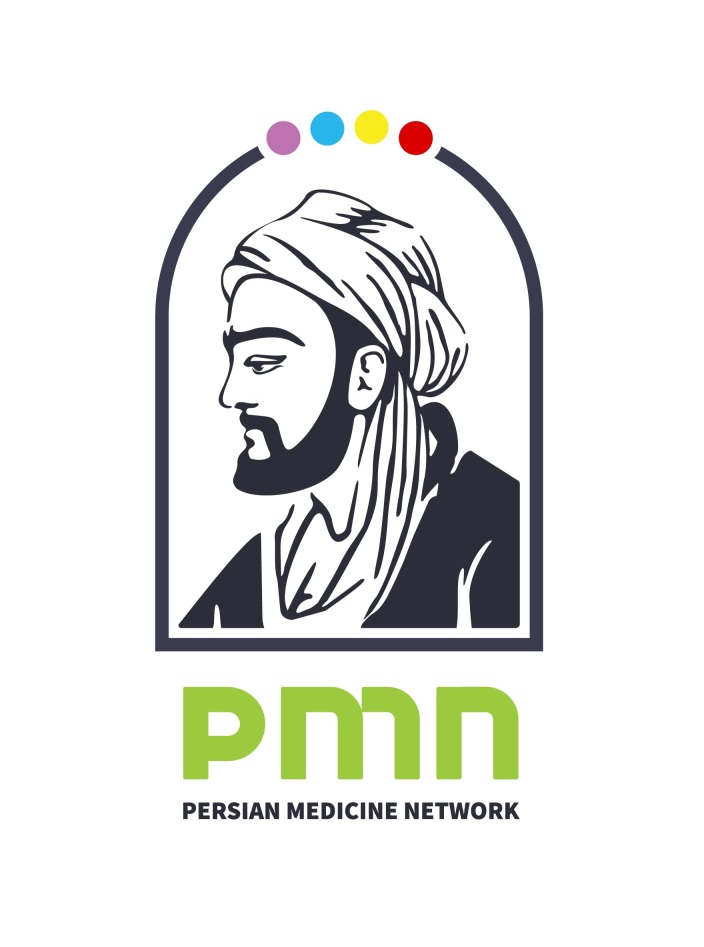
Figure-1. Official logo of Persian Medicine Network (PMN) interest group.

Universal Scientific Education and Research Network (USERN) is a non-profit organization that provides a platform for collaboration and exchange of knowledge among scientists and researchers from different countries. The primary aim of USERN is to promote scientific education and research in all fields of science, including medicine, engineering, and natural sciences. Professor Nima Rezaei, the first president of USERN, founded this organization in 2015. Its headquarter office is located in Children’s Medical Center, Tehran University of Medical Sciences, Tehran, Iran. As an international organization, its creation opened up new opportunities for scientists to expand their knowledge “without border” [1,2]. This has led to the formation of numerous interest groups that allow researchers to concentrate on different scientific fields. The majority of these groups are integrative in nature, which is a notable trait of this expanding global network. This unique attribute has great potential to enhance innovative research endeavors.Although Persian Medicine (PM) has a significant history, it has only been recognized as a distinctive branch of complementary and alternative medicine (CAM) for approximately two decades. As of 2008, Iranian medical universities have begun offering Ph.D. courses in PM and pharmacy for MDs and PharmDs. This marks the start of a scientific movement towards the evidence-based practice of PM.Recent research and educational initiatives have led to a significant increase in scientific publications, resulting in the recognition of PM as a distinct alternative/holistic medical system within international scientific communities. According to a report published in the “Journal of Ethnopharmacology” in 2020, Iran was ranked fifth in the world after China, India, Brazil, and the United States for scientific publications in herbal medicines and ethnopharmacology between 2011 and 2018 [3]. Additionally, Scimago’s report shows that Iran is fourth in the world for scientific production in CAM in 2020 and 2021 [4]. As a result of these achievements, PM has been added to the Medical Subject Headings (MeSH) database of the National Library of Medicine (NLM) since 2022 [5].In the past 20 years, remarkable progress has been made in the field of PM, indicating a promising future. However, it is crucial to continue advancing scientifically while also protecting against fraudulent individuals and pseudoscientific beliefs. To accomplish this, there is a clear need to increase the impact of PM and raise awareness in society. We must collaborate at an international level to improve the quality and quantity of scientific evidence supporting the rational use of PM. The Persian Medicine Network (PMN) (Figure-1) was established within USERN as a community of individuals passionate about PM [6]. Its primary objective is to create a worldwide “science without borders” network that draws upon the expertise and potential of diverse fields to investigate both fundamental and clinical concepts of PM, as well as educational activities.Figure-1. Official logo of Persian Medicine Network (PMN) interest group.

## Conflict of Interest

None.
